# Environmental density, safety, and restoration: a mixed-methods study of behavioral intentions in night-time tourism

**DOI:** 10.3389/fpsyg.2025.1708603

**Published:** 2026-01-12

**Authors:** Sicheng Li, Jingqi Wang

**Affiliations:** 1 Graduate School of Environmental, Life, Natural Science and Technology, Okayama University, Okayama, Japan; 2 School of Business Administration, Dongbei University of Finance and Economics, Dalian, China

**Keywords:** behavioral intention, emotional experience, environmental density, night-time tourism, perceived restorativeness, perceived safety

## Abstract

**Introduction:**

Drawing on Stress Recovery Theory, this study examines how environmental density influences tourists’ behavioral intentions in night-time tourism contexts by integrating the Stimulus-Organism-Response model, Affective Events Theory, and Attention Restoration Theory.

**Methods:**

Using a mixed-methods design, this study analyzes survey data from 653 tourists through Structural Equation Modeling and fuzzy-set Qualitative Comparative Analysis, complemented by field interviews.

**Results:**

The results show that environmental density directly weakens behavioral intention and indirectly affects it through a sequential pathway of perceived safety and emotional experience. Perceived restorativeness significantly buffers the negative effect of density on perceived safety. fsQCA reveals multiple equivalent configurations leading to high and low behavioral intention.

**Discussion:**

The findings extend Stress Recovery Theory to night-time tourism contexts and highlight the importance of density management, safety enhancement, and restorative environmental design.

## 1 Introduction

In recent years, night-time tourism has rapidly expanded globally as a vital component of urban tourism and cultural consumption (World Tourism Organization, 2021). Fueled by accelerated urbanization and diversified tourism consumption patterns, an increasing number of destinations now view night-time activities as a key strategy to extend visitor stays, boost economic returns, and shape urban identity (Zhu and Li, 2025; Khairani et al., 2025). International case studies demonstrate that night-time tourism significantly enhances visitor experiences, stimulates cultural consumption, and boosts destination competitiveness, from large-scale European light festivals (e.g., Lyon’s Festival of Lights) to Asian heritage district night tours (e.g., Kyoto’s Gion Night Festival, Chengdu’s Kuan-Zhai Alley Night Market) (Edensor, 2012; Smith, 2012). However, the environmental context of night-time tourism differs significantly from daytime settings. Factors such as diminished lighting, spatial structural constraints, increased crowd density, and altered perceptions of safety collectively create a complex contextual backdrop influencing visitor psychology and behavior (Himschoot et al., 2024). Social exchange dynamics and emotional solidarity are also shown to influence residents’ support for night tourism, suggesting broader socio-psychological dimensions at play (Wang et al., 2023).

Theoretically, multiple frameworks from environmental psychology and behavioral science provide a robust foundation for analyzing night-time tourism. Among these, the Stimulus-Organism-Response (SOR) model emphasizes the indirect pathway through which external stimuli influence individual psychological states and behaviors (Mehrabian and Russell, 1974). Affective Events Theory (AET) reveals how affective responses mediate between cognitive evaluations and behaviors (Weiss and Cropanzano, 1996), while Attention Restoration Theory (ART) highlights the role of environmental characteristics in alleviating stress and restoring emotional states (Kaplan and Kaplan, 1989). Although these theories have been applied separately in tourism research (Prayag et al., 2017; Ruan et al., 2023; Zhang B. et al., 2023), systematic empirical research integrating all three within night-time tourism contexts remains scarce (Ruiz et al., 2021). From a stress-recovery perspective, existing research has rarely examined how crowd-related environmental stressors function in night-time tourism contexts. By conceptualizing environmental density as a salient environmental stressor and incorporating perceived restorativeness as a buffering mechanism, this study theoretically extends Stress Recovery Theory (SRT) to high-density night-time tourism settings (Ulrich, 1983).

Existing research exhibits shortcomings in three areas: First, while studies confirm Environmental Density (ED) influences tourist perceptions and behaviors, most focus on day-time settings (Eroglu et al., 2005), lacking specific exploration of the unique environmental-psychological-behavioral chain mechanism in night-time tourism. Second, Perceived Safety (PS) and Emotional Experiences (EE) are often tested separately as mediating variables in tourism behavior research (Hosany et al., 2015; Baker and Crompton, 2000), yet their integrated path as a continuous chain mediation has not been validated. Third, Perceived Restorativeness (PR) is widely validated in environmental psychology as a key variable buffering the negative effects of high density or stressors (Korpela et al., 2010; Pasini et al., 2014), yet its moderating role in high-density night-time tourism settings lacks direct empirical support. Furthermore, most studies rely solely on linear analytical tools like Structural Equation Modeling (SEM), overlooking the potential for nonlinear and multipath equivalent mechanisms, thereby limiting theoretical extrapolation and managerial applicability (Rihoux and Ragin, 2009; Fiss, 2011).

Therefore, drawing on SRT, this study theoretically integrates environmental psychology, AET, and ART. It constructs a multi-pathway model with environmental density as the external stimulus, Perceived Safety and Emotional Experience as chain mediators, and Perceived Restorativeness as a contextual moderator. This model aims to reveal the psychological transmission mechanisms in night-time tourism contexts. Methodologically, this study employs a mixed-methods approach combining questionnaire surveys and contextual interviews (Creswell and Clark, 2017), complemented by SEM and Fuzzy-Set Qualitative Comparative Analysis (fsQCA) to simultaneously validate linear relationships and multiple conditional pathway combinations. The core research questions include: (1) How does Environmental Density influence tourist Behavioral Intentions through Perceived Safety and Emotional Experience? (2) Do Perceived Safety and Emotional Experience form a sequential chain of mediation? (3) Can Perceived Restorativeness significantly buffer the negative impact of high Environmental Density on Perceived Safety? (4) Does Environmental Density directly influence Behavioral Intention? By addressing these questions, this study not only fills a research gap in understanding the environment-psychology-behavior linkage mechanism in night-time tourism contexts but also provides theoretical support and empirical evidence for spatial design, contextual creation, and safety management in destination night-time tourism.

## 2 Research review and hypotheses

### 2.1 Environmental density and perceived safety

The relationship between Environmental Density (ED) and Perceived Safety (PS) has garnered attention in both environmental psychology and consumer environment studies. ED refers to an individual’s subjective perception of the number of people and the degree of space occupation in their surroundings (Machleit et al., 1994). High levels of ED are typically associated with feelings of crowding, psychological stress, and reduced satisfaction (Stokols, 1972; Ruiz et al., 2021). However, the effect of crowding on PS may vary across contexts, some studies indicate crowding reduces PS (Pizam and Mansfeld, 2006), while others find that when crowding is interpreted by tourists as a symbol of social vibrancy, its impact may become positive or neutral (Hou and Zhang, 2021). In night-time tourism, environmental structural complexity amplifies the uncertainty and perceived risk associated with crowding (Himschoot et al., 2024), thereby increasing the likelihood of diminished safety perceptions. From a stress-recovery perspective, environmental density can be viewed as a salient crowd-related stressor that undermines tourists’ perceived safety, particularly under night-time conditions. Based on this, the study proposes:

*H1*: Environmental Density (ED) at tourist destinations exerts a significant negative influence on visitors’ Perceived Safety (PS).

### 2.2 Perceived safety and emotional experiences

Perceived Safety (PS) represents tourists’ comprehensive subjective evaluation of personal, property, and psychological security (George, 2010). It has been widely demonstrated to hold a central position in tourism experience and behavioral intention studies (Baker and Crompton, 2000; Qi et al., 2009; Karl et al., 2020). According to AET, cognitive evaluations precede affective responses, meaning that heightened safety perceptions elicit positive emotional reactions (Weiss and Cropanzano, 1996). In the context of tourism, such emotional reactions often manifest as joy, love, and positive surprise, as conceptualized in the Destination Emotion Scale (Hosany et al., 2015).

In this study, Emotional Experience (EE) is conceptualized as a second-order reflective construct comprising Joy, Love, and Positive Surprise. This higher-order specification aligns with emotional appraisal theory and destination emotion research (Prayag et al., 2017), which posit that affective responses in tourism settings are best understood as integrated emotional states rather than isolated discrete emotions. In night-time tourism specifically, PS arises not only from objective conditions such as public order and lighting but also from contextual stimuli such as environmental density, which intensify uncertainty and shape tourists’ emotional experiences. Based on this, the study proposes:

*H2*: Tourists’ Perceived Safety (PS) positively influences their Emotional Experience (EE).

### 2.3 Emotional experience and behavioral intention

Emotional experience (EE) represents tourists’ affective responses to destination contexts and serves as the core psychological driver of Behavioral Intention (BI) (Prayag et al., 2017). Positive emotions not only enhance destination attractiveness but also strengthen intentions to revisit, recommend, and pay a premium (Chen and Tsai, 2007; Su et al., 2017). The Experience Economy Theory posits that emotional value constitutes a vital component of tourism product competitiveness, particularly within the immersive context of night-time tourism (Pine and Gilmore, 2011). Based on this, this study proposes:

*H3*: Tourists’ Emotional Experience (EE) positively influences their Behavioral Intention (BI).

### 2.4 Sequential mediation effect

Within the causal chain linking ED to BI, PS and EE may form a continuous psychological mediating pathway, consistent with the SOR framework’s “stimulus-cognition-emotion-behavior” transmission mechanism (Mehrabian and Russell, 1974). From a stress-recovery lens, this sequential pathway specifies how a crowd-related stressor (ED) translates into behavioral intention via safety evaluation (PS) and subsequent affective responses (EE). Existing research confirms that crowding perception influences tourist satisfaction and behavioral intention through emotional responses (Ruiz et al., 2021). In this study, Emotional Experience (EE) is conceptualized as a second-order affective construct that integrates joy, love, and positive surprise. Thus, its mediating effect represents tourists’ holistic emotional responses rather than individual discrete emotions. Based on this theoretical logic, this study proposes:

*H4a*: Tourists’ Perceived Safety (PS) independently mediates the relationship between Environmental Density (ED) and Behavioral Intention (BI).

*H4b*: Tourists’ Emotional Experience (EE) independently mediates the relationship between Perceived Safety (PS) and Behavioral Intention (BI).

*H4c*: Perceived Safety (PS) and Emotional Experience (EE) jointly form a sequential mediating pathway between Environmental Density (ED) and Behavioral Intention (BI).

### 2.5 The moderating role of perceived restorativeness

From a stress-recovery perspective, Perceived Restorativeness (PR) represents a critical recovery resource that enables individuals to buffer or offset the detrimental effects of environmental stressors. It refers to an individual’s perception of an environment’s capacity to relax, restore attention, and alleviate stress (Kaplan and Kaplan, 1989; Pasini et al., 2014). ART emphasizes that highly restorative environments can buffer the negative effects of external stressors (e.g., high density). In night-time tourism, high PR may mitigate the inhibitory effect of high ED on PS (Zhang J. et al., 2023). Therefore, this study proposes:

*H5*: Tourists’ Perceived Restorativeness (PR) significantly attenuates the negative impact of Environmental Density (ED) on their Perceived Safety (PS), meaning this negative effect weakens when PR is high.

### 2.6 Direct effects of environmental density on behavioral intentions

Environmental Density (ED) has long been identified as a direct driver of avoidance-oriented behaviors. Early environmental psychology shows that high density induces stress and loss of perceived control (Stokols, 1972), while servicescape research finds that crowding directly reduces satisfaction and approach intentions (Eroglu et al., 2005). Meta-analytic evidence further confirms that spatial crowding typically leads to unfavorable customer outcomes (Blut and Iyer, 2020), suggesting that ED can influence behavior even without cognitive or affective mediation.

In tourism contexts, crowding has been shown to diminish experience quality (Sanz-Blas et al., 2024), decrease loyalty in heavily visited areas (Nie et al., 2022), and reduce revisit intentions in crowded service settings (Cakici et al., 2021). ED is increasingly conceptualized as comprising both human and spatial components (Mehta, 2013), a distinction crucial for night-time tourism where limited visibility and heightened safety concerns amplify density perceptions. Under such conditions, high ED is especially likely to trigger avoidance responses such as shortened stays or reduced willingness to revisit. Based on this evidence, this study proposes:

*H6*: Environmental Density (ED) exerts a significant negative direct effect on tourists’ Behavioral Intentions (BI).

### 2.7 Research model

**Figure 1:**
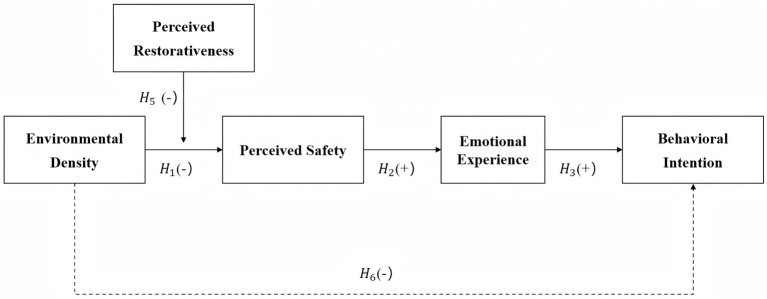
Conceptual research model. Conceptual model showing the relationships among Environmental Density, Perceived Safety, Emotional Experience, Behavioral Intention, and the moderating role of Perceived Restorativeness.

This study integrates Environmental Psychology, AET, and ART to form the research model depicted in Figure 1: Environmental Density (ED) of the destination serves as an external stimulus, influencing Behavioral Intention (BI) through the sequential mediating effects of visitors’ Perceived Safety (PS) and Emotional Experiences (EE). The model incorporates the moderating role of Perceived Restorativeness (PR), while also accounting for the direct path from ED to BI.

## 3 Research design and methodology

### 3.1 Variable measurement

All latent variables in this study employed internationally validated scales, with contextual adaptations tailored to night-time tourism settings. Preserving original semantics and structure, all items utilized a 7-point Likert scale (1 = “Strongly Disagree,” 7 = “Strongly Agree”) to ensure comparability and cross-cultural applicability of quantitative data.

#### 3.1.1 Environmental density (ED)

ED measurement is based on the classic Crowding Perception Scale (Machleit et al., 1994). It encompasses two dimensions: Perceived Human Crowding and Perceived Spatial Crowding, each comprising 4 items for a total of 8 items. This scale has been widely applied in retail, festival, and tourism settings, with empirical research demonstrating robust reliability and construct validity (Stemmer et al., 2024).

#### 3.1.2 Perceived safety (PS)

PS employs George’s (2010) Tourist Perceptions of Safety and Security (TPSS) Scale, comprising 12 items covering dimensions such as accommodation, dining, shopping, public safety, and lighting. This scale is widely used in tourism safety perception research and has demonstrated high reliability and good validity across diverse cultural contexts (Zou and Yu, 2022).

#### 3.1.3 Emotional experience (EE)

EE is measured using the Destination Emotion Scale (DES) developed by Hosany et al. (2015), which comprises three first-order emotional dimensions, Joy, Love, and Positive Surprise, consisting of 15 items in total. Following Hosany et al. (2015) and Prayag et al. (2017), this study conceptualizes EE as a second-order reflective construct, in which the three first-order emotions load onto a higher-order latent factor representing tourists’ overall emotional experience. This specification aligns with emotional appraisal theory, which views affective responses in tourism settings as integrated emotional states rather than isolated discrete emotions. The scale has demonstrated high convergent and discriminant validity and strong cross-cultural adaptability (Prayag et al., 2017).

#### 3.1.4 Behavioral intention (BI)

BI was measured using Chen and Tsai's (2007) scale, comprising 5 items covering dimensions such as revisit intention, recommendation intention, and willingness to pay a premium. This scale exhibits high reliability in tourism behavior research and has been repeatedly validated as an effective predictor of subsequent tourist decision-making.

#### 3.1.5 Perceived restorativeness (PR)

PR is measured using the PRS-11 Scale proposed by Pasini et al. (2014), comprising 11 items across four dimensions: Fascination, Being Away, Coherence, and Scope. This scale has demonstrated stable reliability and good cross-cultural applicability in environmental psychology (Korpela et al., 2018).

To ensure semantic equivalence and cultural appropriateness, all measurement items originally developed in English were translated into Chinese following Brislin’s (1980) back-translation procedure. First, two bilingual researchers independently translated the items into Chinese. Second, another bilingual expert, who had not seen the original scales, back-translated the Chinese items into English. Discrepancies were reviewed and resolved through panel discussion to ensure conceptual consistency. Finally, a pilot test with 30 participants was conducted to assess clarity, readability, and contextual relevance in night-time tourism settings, leading to minor wording refinements.

### 3.2 Data collection methods and sample

This study employs a mixed-methods design combining quantitative and qualitative approaches (Creswell and Clark, 2017). This design enables complementary hypothesis testing via SEM and exploratory configuration analysis through fsQCA, supplemented by contextual interpretations from semi-structured interviews.

For the quantitative survey, questionnaires were distributed online via the “Wenjuanxing platform” (a Chinese online survey platform) and targeted through social media, travel forums, and interest communities to screen respondents with night-time tourism experiences within the past year. To ensure structural balance and representativeness, stratified random sampling was employed, allocating samples by gender, age, and proportion of night-time tourism experience. A total of 698 questionnaires were collected. After excluding incomplete or invalid responses, 653 valid samples were obtained (valid response rate: 93.55%).

For the qualitative component, the research team conducted semi-structured interviews during fieldwork visits to renowned night-time tourist attractions in Dalian and Chengdu of China. Using random intercept sampling, visitors were approached on-site and invited to share their experiences. The interviews focused on themes such as environmental density, perceived safety, emotional experiences, and behavioral intentions. Respondents were further encouraged to reflect on how specific environmental characteristics of night-time tourism settings shaped their psychological responses and behavioral tendencies.

This study followed standard ethical guidelines for human-subject research. All participants were informed of the study purpose, assured of anonymity, and provided informed consent prior to participation. Participation was entirely voluntary, and respondents had the right to withdraw at any time. According to institutional policies for minimal-risk survey research, the study met the criteria for ethical exemption and therefore did not require formal ethical committee approval.

### 3.3 Data analysis methods

Data analysis employed a multi-stage, multi-method strategy encompassing reliability and validity testing, common method bias assessment, discriminant validity testing, SEM, and fsQCA.

In the first stage, SPSS 24.0 was used for data cleaning and measurement evaluation, including descriptive statistics, internal consistency reliability (Cronbach’s *α*), Composite Reliability (CR), and Average Variance Extracted (AVE). Discriminant validity was assessed using the Fornell–Larcker criterion (Fornell and Larcker, 1981), supplemented by the HTMT test with a threshold of 0.85 (Henseler et al., 2015). Common method bias risk was assessed using the Harman single-factor test (Podsakoff et al., 2003). To further minimize the risk of common method bias, the full collinearity VIF technique recommended by Kock (2015) was also applied. All latent variables (ED, PS, EE, PR) were regressed simultaneously in an ordinary least squares (OLS) model using IBM SPSS 24.0 to obtain collinearity diagnostics. The resulting VIF values ranged from 1.17 to 2.10, all well below the recommended threshold of 3.3, indicating that common method bias was not a serious concern. Additionally, normality of variable distributions was examined via Q-Q plots, observing the fit of data points to the theoretical normal distribution’s 45° diagonal line to ensure compliance with SEM’s normality assumption.

In the second stage, Confirmatory Factor Analysis (CFA) and SEM were conducted using AMOS 24.0. Model fit was assessed according to Hu and Bentler's (1999) recommendations: *χ*^2^/df < 3.0, CFI ≥ 0.90, TLI ≥ 0.90, RMSEA ≤0.08. The structural model was first used to test direct path effects, with hypothesis validity determined by standardized path coefficients (*β*) and their significance levels (*p*-values). Subsequently, indirect effects and their 95% confidence intervals were estimated using Bootstrap method with 5,000 resamples (Preacher and Hayes, 2008) to validate the significance and robustness of single-mediation and sequential-mediation paths. Moderation testing was conducted concurrently using Multi-Group Analysis (MGA) and interaction terms: first, moderator variables were grouped based on mean ± 1 standard deviation to compare significant differences in path coefficients between high and low groups; then, centered interaction terms were introduced to observe their incremental explanatory power (Δ*R*^2^) and significance for the dependent variable.

Finally, this study used fsQCA 3.0 to reveal the configurational effects of multiple condition combinations on high BI (Fiss, 2011). After setting full membership points, crossover points, and full non-membership points via direct calibration, perform necessity and sufficiency analyses to extract configuration paths meeting both consistency (consistency ≥0.80) and raw coverage (raw coverage ≥0.10) thresholds. This step complements SEM’s assumption limitations on linear relationships, aids in revealing nonlinear mechanisms and multiple equivalent pathways, and provides cross-methodological validation for BI formation under different condition combinations.

## 4 Empirical analysis

This chapter systematically presents data analysis results along the validation pathway of the research model, sequentially covering sample characteristic distributions; descriptive statistics and preliminary correlation tests for latent variables; measurement model reliability and validity testing; structural path analysis; mediation effect analysis; fsQCA configuration path identification; and robustness and common method bias tests. By integrating quantitative methods with configurational analysis, this chapter aims to comprehensively reveal the operational mechanisms through which ED influences BI, laying an empirical foundation for subsequent theoretical discussions and practical recommendations.

### 4.1 Descriptive statistics and correlation analysis

#### 4.1.1 Sample characteristic analysis

To ensure data representativeness and analytical rigor, this study statistically analyzed the basic sociodemographic characteristics and night-time tourism preference types of 653 valid samples. Dimensions included gender, age, education level, occupation type, annual income, and night-time tourism activity types (Table 1).

**Table 1:** Profile of respondents and night tourism type (*N* = 653).

Variable	Category	Frequency	Percentage
Gender	Female	350	53.6
	Male	303	46.4
Age	18–25	139	21.3
	26–35	189	28.9
	36–45	155	23.8
	46+	170	26.0
Education	Associate degree	146	22.3
	Bachelor’s degree	325	49.8
	High school or below	62	9.5
	Master’s or above	120	18.4
Occupation	Enterprise employee	219	33.5
	Freelancer	105	16.1
	Government staff	85	13.0
	Other	56	8.6
	Retired	29	4.4
	Student	159	24.4
Annual income	100 k–200 k	88	13.5
	30 k–60 k	126	19.3
	60 k–100 k	177	27.1
	<30 k	223	34.1
	>200 k	39	6.0
Night tourism type	Urban night view sightseeing	188	28.8
	Historic and cultural districts	50	7.7
	Other	32	4.9
	Commercial street night-time shopping	127	19.4
	Cultural performances, festivals, and lighting events	59	9.0
	Night market and culinary	135	20.7
	Theme park nightclubs	62	9.5

This table summarizes the demographic information (e.g., gender, age, education, occupation, income) and Night Tourism Type of the 653 valid respondents. Percentages are rounded to one decimal place.

Regarding gender distribution, females accounted for 53.6% of the sample while males constituted 46.4%, indicating a relatively balanced gender ratio. In terms of age, respondents predominantly fell within the 26–35 (28.9%) and 36–45 (23.8%) age brackets, which together exceeded half of the total sample, highlighting that young and middle-aged individuals form the primary participant group for night-time tourism. Regarding educational attainment, nearly half of respondents held bachelor’s degrees (49.8%), while those with master’s degrees or higher accounted for 18.4%. A total of 68.2% possessed bachelor’s degrees or higher, indicating a high overall educational level among the sample. Occupationally, enterprise employees (33.5%) and students (24.3%) constituted significant proportions, reflecting diverse professional backgrounds. Annual income distribution was dominated by “<30 k” (34.1%) and “60 k-100 k” (27.1%), with a higher proportion of low-to-middle income groups, making the overall sample reasonably representative in economic terms. Regarding night-time tourism preferences, “Urban Night View Sightseeing” ranked highest (28.8%), followed by “Night Market and Culinary” (20.7%) and “Commercial Street Night-time Shopping” (19.4%), indicating consumers favor visual experiences and consumption-oriented night-time activities. Cultural experiences like “Historic and Cultural Districts” (7.7%) and “Cultural Performances, Festivals, and Lighting Events” (9.0%) were selected less frequently, indicating that leisure and entertainment remain dominant drivers of night-time tourism behavior.

#### 4.1.2 Descriptive statistics analysis of latent variables

This study further conducted descriptive statistical analysis on five core latent variables: Environmental Density (ED), Perceived Safety (PS), Emotional Experience (EE), Behavioral Intention (BI), and Perceived Restorativeness (PR) (Table 2). Results indicate that the mean value for each variable is 4.00, positioned at the midpoint of the 7-point Likert scale. This reflects respondents’ generally neutral evaluations of these dimensions, with no pronounced extreme tendencies. Standard deviations for all variables range between 1.30 and 1.44, suggesting a moderate dispersion of opinions across constructs within the sample. This adequate variability supports subsequent inferential analysis.

**Table 2:** Descriptive statistics of latent constructs (ED, PS, EE, BI, PR).

Variable	Mean	Std. Dev.	Min	Max
ED	4.00	1.44	1.00	7.00
PS	4.00	1.37	1.00	7.00
EE	4.00	1.30	1.20	6.87
BI	4.00	1.44	1.00	7.00
PR	4.00	1.41	1.00	7.00

This table presents the mean, standard deviation, minimum, and maximum values of the five core latent constructs. All constructs are measured on a 7-point Likert scale, and each composite score is calculated as the average of its respective items. ED, Environmental Density; PS, Perceived Safety; EE, Emotional Experience; BI, Behavioral Intention; PR, Perceived Restorativeness.

Regarding extremes, all variables exhibited a minimum value of 1.00 and a maximum value close to 7.00 (EE variable peaked at 6.87), indicating comprehensive response coverage across the scale’s extremes without significant ceiling or floor effects. Despite the mean’s midpoint position, the variables retained sufficient variance information for advanced analyses like SEM.

**Figure 2:**
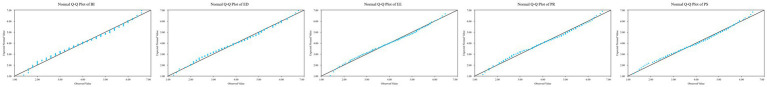
Q-Q plots for latent variables. Q-Q plots of Environmental Density (ED), Perceived Safety (PS), Emotional Experience (EE), Perceived Restorativeness (PR), and behavioral intention (BI), illustrating that sample quantiles align closely with the 45° reference line, supporting the assumption of normality.

Additionally, as shown in the Q-Q plot results in Figure 2, the sample quantiles of the five latent variables (ED, PS, EE, BI, PR) largely follow the 45° reference line, with only slight deviations at the tails. This indicates an approximately normal distribution without significant ceiling or floor effects, meeting the assumption for maximum likelihood estimation. For robustness, the empirical analysis also reports robust standard errors and bootstrap confidence intervals.

### 4.2 Measurement model evaluation

To ensure the reliability and validity of latent variable measurement tools and the fit of the theoretical model, this study employed CFA under SEM to systematically examine five core constructs (ED, PS, EE, BI, PR). The evaluation encompassed convergent validity, discriminant validity, and overall model fit indices. In line with the theoretical specification, Emotional Experience (EE) was modeled as a second-order reflective construct composed of three first-order emotional dimensions (Joy, Love, and Positive Surprise).

#### 4.2.1 Convergent validity

All standardized factor loadings for latent variables exceeded 0.50 (Table 3), indicating that each item effectively reflected its assigned latent structure. Table 3 also shows that all variables’ AVE exceeded 0.50 and CR surpassed the 0.70 threshold, confirming the scale meets ideal statistical standards for convergent validity and internal consistency. This indicates the measurement tools for each latent variable possess strong convergent explanatory power and measurement stability, providing reliability assurance for subsequent structural path estimation.

**Table 3:** Measurement model evaluation results.

Construct	AVE	CR	Item/Dimension	Standardized loading
Environmental density	0.614	0.927	ED1	0.834
			ED2	0.802
			ED3	0.799
			ED4	0.745
			ED5	0.836
			ED6	0.783
			ED7	0.762
			ED8	0.748
Perceive safety	0.566	0.939	PS1	0.819
			PS2	0.831
			PS3	0.797
			PS4	0.79
			PS5	0.793
			PS6	0.742
			PS7	0.738
			PS8	0.749
			PS9	0.705
			PS10	0.741
			PS11	0.721
			PS12	0.706
Emotional experience	0.507	0.938	Joy (first-order)	0.891
			J1	0.784
			J2	0.731
			J3	0.716
			J4	0.68
			J5	0.664
			Love (first-order)	0.891
			L1	0.773
			L2	0.732
			L3	0.726
			L4	0.685
			L5	0.657
			Positive Surprise (first-order)	0.907
			P1	0.76
			P2	0.774
			P3	0.717
			P4	0.691
			P5	0.699
Behavioral intention	0.579	0.872	BI1	0.806
			BI2	0.799
			BI3	0.743
			BI4	0.749
			BI5	0.728
Perceived restorativeness	0.597	0.942	PR1	0.85
			PR2	0.829
			PR3	0.813
			PR4	0.795
			PR5	0.759
			PR6	0.76
			PR7	0.766
			PR8	0.784
			PR9	0.761
			PR10	0.721
			PR11	0.74

All factor loadings are standardized and statistically significant (*p* < 0.001). AVE = average variance extracted; CR = composite reliability. For Emotional Experience (EE), standardized second-order factor loadings of the first-order dimensions (Joy, Love, and Positive Surprise) are reported above the corresponding item-level loadings. All AVE and CR values exceed the recommended thresholds, indicating satisfactory convergent validity and internal consistency.

#### 4.2.2 Discrimination validity testing

This study further employed the Fornell–Larcker criterion to assess discriminant validity among variables. Table 4 shows that the square root of AVE (diagonal) for all constructs exceeded their respective correlation coefficients with other constructs (off-diagonal). While most correlations were significant, none exceeded the multicollinearity threshold (*r* < 0.85). Thus, the latent variables in this study demonstrate statistically sound structural independence, with no significant risk of overlapping between variables.

**Table 4:** Discriminant validity of constructs: Fornell–Larcker criterion.

Construct	ED	PS	EE	BI	PR
ED	0.784				
PS	−0.524	0.753			
EE	−0.288	0.564	0.712		
BI	−0.393	0.528	0.583	0.761	
PR	−0.059	0.351	0.176	0.157	0.773

Values on the diagonal represent the square root of AVE; off-diagonal values are Pearson correlations between constructs. Discriminant validity is established when the square root of AVE (in bold) exceeds inter-construct correlations. ED, Environmental Density; PS, Perceived Safety; EE, Emotional Experience; BI, Behavioral Intention; PR, Perceived Restorativeness.

#### 4.2.3 Model fit analysis

To further examine the overall validity of the measurement model, AMOS 24.0 was used to calculate model fit indices, including standard metrics. As shown in Table 5, χ^2^/df = 1.736, well below the empirically recommended value of 3; RMSEA = 0.034, indicating excellent residual structural fit; CFI, TLI, and IFI were 0.959, 0.957, and 0.959 respectively, all substantially exceeding the 0.90 threshold, indicating excellent model fit. Therefore, the measurement model demonstrates high overall structural fit, satisfying the statistical prerequisites for proceeding to structural path analysis. In addition, a hierarchical CFA was conducted to compare a first-order model and a second-order model for EE. The second-order specification yielded comparable or better fit indices, and all second-order factor loadings were significant (*p* < 0.001), further supporting the validity of treating EE as a higher-order affective construct.

**Table 5:** Model fit indices for the measurement model.

Commonly used indicators	χ^2^*/df*	RMSEA	CFI	TLI	IFI
Results	1.736	0.034	0.959	0.957	0.959
Judgment criteria	<3	<0.08	>0.9	>0.9	>0.9
Reasonable or not	Yes	Yes	Yes	Yes	Yes

*χ*^2^/df = Chi-square divided by degrees of freedom; RMSEA, Root Mean Square Error of Approximation; CFI, Comparative Fit Index; TLI, Tucker–Lewis Index; IFI, Incremental Fit Index. All indices meet the recommended thresholds, indicating good model fit.

In summary, the combined results of convergent validity, discriminant validity, and model fit assessments demonstrate that the measurement tools in this study exhibit good alignment between conceptual dimension construction and empirical data performance. The theoretical structure has been empirically validated, providing a solid foundation for in-depth examination of structural pathways and mediating mechanisms.

#### 4.2.4 Common method bias examination

Given the self-report nature of the questionnaire data, this study examined CMB risks. Harman’s single-factor test revealed that the first factor explained 31.267% of the variance, below the 40% critical threshold (Podsakoff et al., 2003), indicating no severe single-factor bias. Furthermore, a CFA model loading all measurement items onto a single latent variable yielded significantly poorer fit indices than the measurement model (CFI/TLI decreased significantly, RMSEA increased), indicating the single-factor model was not adequate. In addition, the full collinearity VIFs for all latent constructions ranged from 1.17 to 2.10, well below the threshold of 3.3 (Kock, 2015), which further suggests that common method bias was not a serious concern in this study. Thus, it can be concluded that the data in this study were not subject to severe common method bias.

### 4.3 Structural model results

Following validation of the measurement model via confirmatory factor analysis, this study constructed a structural path model to explore the complex mechanisms through which ED influences BI.

#### 4.3.1 Path coefficients and significance tests

Path analysis examined the mechanisms linking ED, PS, EE, and BI. Standardized path coefficients and significance test results are presented in Table 6.

**Table 6:** Standardized path coefficients and significance test results.

Path	Standardized path coefficients	*z*-value (CR)	*p*-value	Significance
ED → PS	−0.524	−15.719	***	Significant
ED → EE	0.010	0.270	0.787	Not Significant
PS → EE	0.569	15.009	***	Significant
ED → BI	−0.164	−4.686	***	Significant
PS → BI	0.204	5.026	***	Significant
EE → BI	0.421	11.648	***	Significant

All coefficients are standardized estimates. CR = Critical Ratio (*z*-value); ***p < 0.001, **p < 0.01, *p < 0.05. Significance is determined based on the *z*-value and *p*-value. Non-significant paths indicate lack of direct effect. ED, Environmental Density; PS, Perceived Safety; EE, Emotional Experience; BI, Behavioral Intention; PR, Perceived Restorativeness.

The analysis revealed that ED significantly negatively influenced PS (*β* = −0.524, *p* < 0.001), indicating that increased crowd density markedly reduced individuals’ sense of security. Concurrently, ED exerted a significant direct negative effect on BI (*β* = −0.164, *p* < 0.001). However, ED’s direct effect on EE was not significant (*β* = 0.010, *p* = 0.787), indicating that environmental density is not a primary direct predictor of emotional responses.

PS significantly and positively influenced EE (*β* = 0.569, *p* < 0.001) and also significantly and positively promoted BI (*β* = 0.204, *p* < 0.001). EE exerted the most significant influence on BI (*β* = 0.421, *p* < 0.001), indicating that positive emotional responses are a key driver of behavioral willingness.

Overall, PS and EE play crucial mediating roles between ED and BI. ED exerts a partial direct negative effect on BI, while its influence on EE occurs indirectly through PS.

#### 4.3.2 Structural model diagram

**Figure 3:**
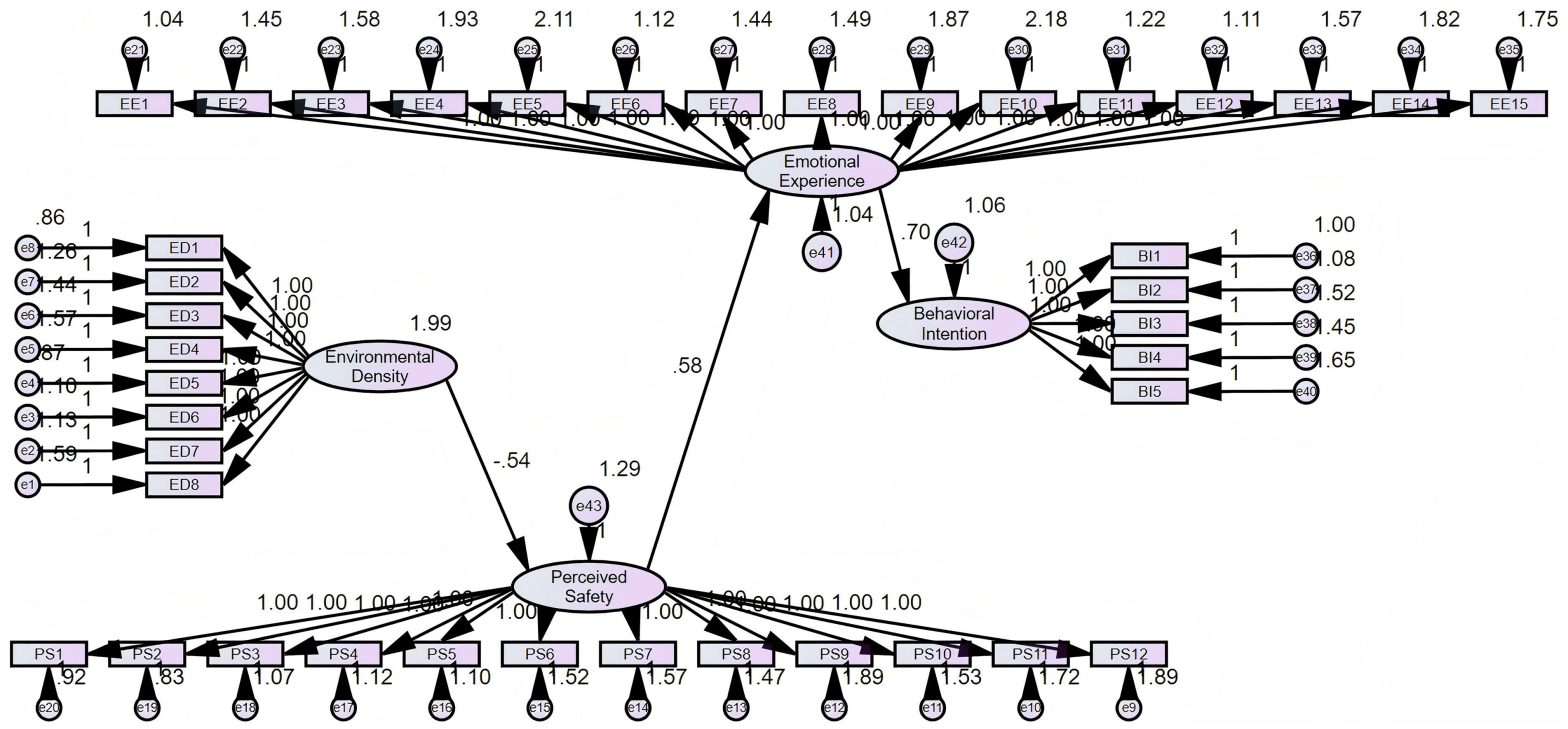
Structural equation model results. Standardized path coefficients of the structural model. Environmental Density negatively affects Perceived Safety, which positively predicts Emotional Experience and Behavioral Intention. Emotional Experience also strongly predicts Behavioral Intention.

To aid understanding of path relationships, this study developed a standardized structural path diagram (Figure 3) that visually depicts the primary pathway and mediating network: ED → PS → EE → BI. All paths in the diagram are labeled with standardized regression coefficients (*β*) and significance levels, clearly illustrating the progressive logical mechanism from ED to BI.

In summary, the structural path model confirms that the influence of ED on BI is not a simple direct relationship. Instead, it involves a sequential mediation pathway with PS and EE as key mediating variables, collectively forming a complex transmission mechanism. Specifically, ED not only directly negatively influences BI but also indirectly weakens EE by reducing PS, ultimately inhibiting BI formation. This demonstrates ED’s multi-layered mode of action on BI.

### 4.4 Multi-group robustness analysis

To assess the robustness of the proposed structural relationships, split-sample structural equation modeling analyses were conducted across gender, age, and education level, with separate models estimated for each subgroup. Table 7 presents the results of the multi-group analyses across gender, age, and education groups.

**Table 7:** Multi-group analysis results (standardized path coefficients).

Path	Gender (Male/Female)		Age group (Low/High)		Education level (Low/High)	
	Male	Female	Low	High	Low	High
ED → PS	−0.549***	−0.503***	−0.514***	−0.534***	−0.527***	−0.522***
PS → EE	0.553***	0.572***	0.550 ***	0.577 ***	0.456***	0.611***
EE → BI	0.598***	0.573***	0.570 ***	0.596***	0.576***	0.586***
ED → BI	−0.392***	−0.394***	−0.373 ***	−0.413 ***	−0.378***	−0.397***

Values are standardized path coefficients obtained from split-sample structural equation modeling analyses. Separate models were estimated for gender, age, and education subgroups. ***p < 0.001.

The results show a high degree of consistency in both the magnitude and direction of the estimated path coefficients across all demographic categories. Environmental density exhibits a stable negative association with perceived safety across gender (*β* = −0.503 to −0.549), age (*β* = −0.514 to −0.534), and education groups (*β* = −0.522 to −0.527), as well as a consistent negative effect on behavioral intention (*β* = −0.373 to −0.413).

In contrast, perceived safety positively influences emotional experience across all subgroups, with standardized coefficients ranging from 0.553 to 0.572 (gender), 0.550 to 0.577 (age), and 0.456 to 0.611 (education). Emotional experience, in turn, shows a robust positive association with behavioral intention across demographic categories (*β* = 0.570 to 0.598 for gender and age; *β* = 0.576 to 0.586 for education). All path coefficients remain statistically significant at *p* < 0.001.

Overall, the consistency of both significance levels and coefficient directions across demographic subgroups indicates that the proposed structural relationships are robust to variations in gender, age, and educational background.

### 4.5 Bootstrap analysis

To systematically elucidate the mechanism of ED’s influence on BI, this study employed a Bootstrap method based on 5,000 resamples. Using the PROCESS plugin (Model 6 and Model 7), the significance and effect strength of the sequential mediating path and moderating effects were separately examined.

#### 4.5.1 Sequential mediation effect analysis: ED → PS → EE → BI

To further validate the hierarchical transmission structure of the mediation mechanism, Model 6 examined the sequential path ED → PS → EE → BI. Results (Table 8) indicate: Bootstrap mediation tests revealed a significant negative total effect of ED on BI. This influence operates through two pathways: first, a direct negative effect of ED on BI; second, a sequential path mediated by PS (ED → PS → EE → BI), wherein Environmental Density reduces tourists’ Perceived Safety, thereby diminishing their Emotional Experience and ultimately inhibiting Behavioral Intention. Among these, Perceived Safety (PS) serves as the most central mediating variable. It independently mediates the effect of ED on BI and also forms a sequential mediation pathway by influencing EE. Notably, the independent mediating effect of EE was not significant when separated from the sequential pathway involving PS, indicating that Emotional Experience contributes to Behavioral Intention primarily through its position within the “ED → PS → EE → BI” chain. This sequential mediating pathway exhibits the largest effect size, representing the core mechanism that validates the sequential mediating role of perceived safety and emotional experience between crowded environments and behavioral decisions.

**Table 8:** Bootstrap results for direct, indirect, and total effects.

Path	Coeff	Boot SE	*p*	95%CI	Significance
Total effect	−0.3928	0.0361	0	[−0.4636, −0.3220]	√
Direct effect	−0.1644	0.0352	0	[−0.2334, −0.0953]	√
Indirect effect	−0.2284	0.0273		[−0.2842, −0.1748]	√
ED → PS → BI	−0.107	0.022		[−0.1516, −0.0645]	√
ED → EE → BI	0.0042	0.0159		[−0.0271, 0.0357]	×
ED → PS → EE → BI	−0.1256	0.0158		[−0.1583, −0.0959]	√

This table presents the estimates from a serial mediation analysis using PROCESS Model 6. Bootstrapped standard errors and 95% confidence intervals (CIs) based on 5,000 resamples are reported. Significance is indicated by whether the 95% CI excludes zero (√ = significant, × = not significant). ED, Environmental Density; PS, Perceived Safety; EE, Emotional Experience; BI, Behavioral Intention; PR, Perceived Restorativeness.

#### 4.5.2 Moderating effect analysis

This study employed moderation analysis to examine the moderating role of Perceived Restorativeness (PR) in the relationship between Environmental Density (ED) and Perceived Safety (PS). As shown in Table 9, the interaction term between ED and PR (ED × PR) exerted a significant positive effect on PS (*β* = 0.1450, t = 6.939, *p* < 0.001, 95% CI [0.1039, 0.1860]), indicating that PR significantly moderated the effect of ED on PS.

**Table 9:** Coefficients for main effects and interaction term.

Type of effect	Coeff.	SE	*t*	*p*	95%CI	Significance
Constant	4.0173	0.0411	97.797	0	[3.9366, 4.0980]	
ED	−0.486	0.0285	−17.056	0	[−0.5420, −0.4301]	√
PR	0.3102	0.0291	10.654	0	[0.2531, 0.3674]	√
ED*PR	0.1450	0.0209	6.939	0	[0.1039, 0.1860]	√

ED, Environmental Density; PR, Perceived Restorativeness.

**Figure 4:**
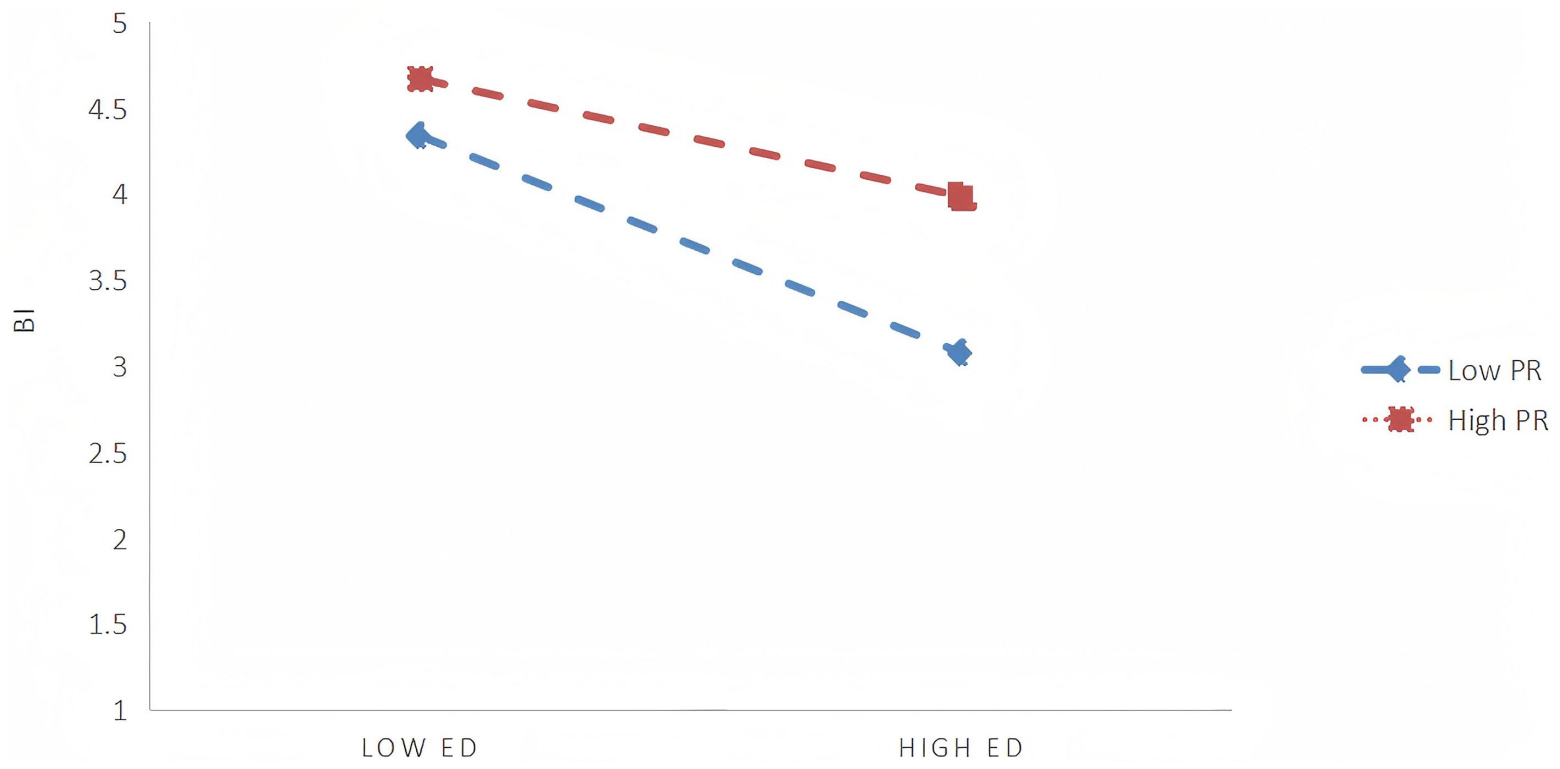
Moderating effect of Perceived Restorativeness. Simple slope analysis showing that Perceived Restorativeness buffers the negative effect of Environmental Density on Behavioral Intention. At high PR levels, the decline in BI under high density is weaker than at low PR levels.

To further elucidate the specific pattern of this moderation, a simple slope analysis was conducted (Table 10; Figure 4). Results revealed significant differences in the intensity of ED’s negative impact on PS across varying PR levels: When PR was at a low level (-1SD), ED exerted its strongest negative effect on PS (*β* = −0.6907, *p* < 0.001); at the mean level of PR, the negative impact was moderate (*β* = −0.4860, *p* < 0.001); and at the higher level of PR (+1SD), the negative impact of ED on PS was weakest (*β* = −0.2814, *p* < 0.001). This indicates that higher Perceived Restorativeness effectively buffers the negative effects of environmental crowding on Perceived Safety.

**Table 10:** Conditional effects of the focal predictor at values of the moderators.

PR	X’s effect	SE	*t*	*p*	95%CI	Significance
−1.4117	−0.6907	0.0415	−16.63	0	[−0.7722, −0.6091]	√
0	−0.486	0.0285	−17.06	0	[−0.5420, −0.4301]	√
1.4117	−0.2814	0.0405	−6.95	0	[−0.3609, −0.2019]	√

The figure shows the interaction effect of ED and PR on PS. Conditional effects of ED on PS are plotted at low (−1 SD), mean, and high (+1 SD) levels of PR. ED, Environmental Density; PS, Perceived Safety; PR, Perceived Restorativeness.

In summary, Perceived Restorativeness plays a positive protective moderating role in the relationship between Environmental Density and Perceived Safety. That is, the higher an individual’s perception of environmental restorativeness, the weaker the negative impact of environmental crowding on their sense of safety.

#### 4.5.3 Analysis of moderated mediation effects

This study further examined whether the moderating effect of Perceived Restorativeness (PR) significantly influenced the entire mediation pathway through a moderated mediation model. As shown in Table 11, both index of moderated mediation reached statistical significance. Specifically, the moderated mediation index via the Perceived Safety (PS) pathway was 0.0311 (BootSE = 0.0078, 95% CI [0.0172, 0.0480]), while the index via the Emotional Experience (EE) pathway was 0.0481 (BootSE = 0.0121, 95% CI [0.0255, 0.0726]). Both Bootstrap confidence intervals excluded zero. This finding indicates that PR not only moderates the direct effects of Environmental Density (ED) on mediating variables (PS and EE), but also significantly moderates the overall strength of the “ED → PS/EE → BI” mediation mechanism. Specifically, the effect size of this mediating pathway systematically varies with individuals’ levels of PR.

**Table 11:** Index of moderated mediation.

Variable	Index	BootSE	95%CI	Significance
PS	0.0311	0.0078	[0.0172, 0.0480]	√
EE	0.0481	0.0121	[0.0255, 0.0726]	√

PS, Perceived Safety; EE, Emotional Experience.

In summary, this study systematically examined the mechanism of ED on BI, the moderating role of PR, and its overall moderation effect on the mediating pathway by integrating path analysis and Bootstrap tests. The results indicate that ED exerts not only a direct negative influence on BI but also an indirect effect through a sequential mediating pathway involving PS and EE, with PS serving as the core mediating variable. Furthermore, PR plays a significant buffering role in the relationship between ED and PS, effectively mitigating the negative impact of environmental crowding on perceived safety. Moderation tests further revealed that PR significantly moderated the strength of the “ED → PS/EE → BI” mediating pathway, indicating that the effectiveness of this mediating mechanism exhibits systematic variations with changes in individual PR levels. In summary, this study unravels the complex mechanisms of multivariable interactions, confirming PR’s protective moderating role in the relationship between crowded environments and visitor behavioral intentions. It provides crucial theoretical foundations and practical insights for understanding and intervening in visitor psychology and behavior within high-density tourism contexts.

#### 4.5.4 Summary of hypothesis testing results

This study systematically tested eight theoretical hypotheses (H1-H6) using SEM and Bootstrap methods, comprehensively examining how ED influences tourist BI through PS and EE, along with the moderating role of PR. The results encompass direct path effects, validation of sequential mediation mechanisms, and moderation effect tests, as detailed in Table 12.

**Table 12:** Summary of hypothesis testing results.

Hypothesis No.	Path relationship	Beta coefficient	Significance
H1	ED → (−) PS	−0.524	Significance
H2	PS → (+) EE	0.569	Significance
H3	EE → (+) BI	0.421	Significance
H4a	ED → PS → BI	−0.107	Significance
H4b	ED → EE → BI	0.004	Not Significance
H4c	ED → PS → EE → BI	−0.123	Significance
H5	PR ⊗ (ED → (−) PS)	0.1450	Significance
H6	ED → (−) BI	−0.164	Significance

ED, Environmental Density; PS, Perceived Safety; EE, Emotional Experience; BI, Behavioral Intention; PR, Perceived Restorativeness.

Results indicate that most research hypotheses are supported by the data. Specifically, ED significantly negatively affects PS (H1: *β* = −0.524, *p* < 0.001), PS significantly positively predicts EE (H2: *β* = 0.569, *p* < 0.001), and EE significantly and positively influenced BI (H3: *β* = 0.421, *p* < 0.001). The mediation analysis revealed that PS played a significant independent mediating role between ED and BI (H4a: *β* = −0.107, 95% CI [−0.1516, −0.0645]). The sequential mediating path ED → PS → EE → BI was also significant (H4c: *β* = −0.123, 95% CI [−0.1583, −0.0959]). However, the independent mediating effect of EE, isolated from the sequential path involving PS, was not significant (H4b: *β* = 0.004, 95% CI [−0.0271, 0.0357]). Furthermore, PR significantly moderated the negative effect of ED on PS (H5: *β* = 0.145, *p* < 0.001), meaning higher PR levels weakened ED’s detrimental impact on PS. The direct negative effect of ED on BI was also significant (H6: *β* = −0.164, *p* < 0.001). In summary, except for the independent mediating effect of EE (H4b), all other hypotheses were supported, indicating that the theoretical model possesses strong explanatory power and revealing the complex causal mechanisms linking ED, PS, EE, PR, and BI.

### 4.6 fsQCA configuration results

This study employs fsQCA to further explore the complex determinants of tourist behavioral intentions in night-time tourism scenarios at the configuration level.

When running and processing data using fsQCA 3.0, the collected data must first undergo calibration. Following the approach of Du et al. (2020), variables were calibrated into fuzzy sets using the direct method. First, referencing Rihoux and Ragin (2009), the full membership point, crossover point, and full non-membership point were set to 0.95, 0.5, and 0.05, respectively. Next, using Excel’s PERCENTILE function, the calibration points for the fuzzy sets of the five variables were calculated (Table 13). Subsequently, the fsQCA3.0 was run to calibrate the variable data using the CALIBRATE function. Since calibrated data included values of 0.5, and fsQCA3.0 does not perform subsequent analysis on such values, this study manually adjusted the 0.5 values to 0.501, following the recommendation proposed by Campbell et al. (2013).

**Table 13:** Fuzzy-set calibration points.

Variables	Fuzzy-set calibration points		
	Full affiliation	Crossover points	Completely unaffiliated
ED	6.375	4.000	1.575
PS	6.117	4.083	1.583
EE	6.133	4.000	1.800
PR	6.273	4.000	1.691
BI	6.400	4.000	1.600

Calculations based on Excel’s PERCENTILE function were compiled by the authors. ED, Environmental Density; PS, Perceived Safety; EE, Emotional Experience; BI, Behavioral Intention; PR, Perceived Restorativeness.

#### 4.6.1 Necessity analysis of individual conditions

Following the standard QCA analytical approach, this study first examined whether individual conditions (including non-sets) are necessary for enhancing BI before conducting sufficiency analysis of condition configurations. Table 14 presents the results of the single-factor necessity analysis for high BI and non-high BI, the consistency levels for all condition variables (including non-sets) are below 0.9. This indicates that BI is determined by multiple factors rather than a single element.

**Table 14:** Necessity analysis of single conditional variable.

Conditional variable	High behavioral intention		Low behavioral intention	
	Consistency	Coverage	Consistency	Coverage
ED	0.568908	0.566837	0.733228	0.726796
~ED	0.725798	0.732245	0.563004	0.565079
PS	0.760813	0.75397	0.549185	0.541442
~PS	0.537281	0.545035	0.750453	0.757362
EE	0.785989	0.781427	0.533648	0.527817
~EE	0.525061	0.530895	0.779013	0.783611
PRS	0.679009	0.677195	0.608957	0.604202
~PRS	0.603141	0.607901	0.674655	0.676476

Organized by the authors based on fsQCA3.0 software run results. ~ denotes “not,” i.e., does not exist. ED, Environmental Density; PS, Perceived Safety; EE, Emotional Experience; BI, Behavioral Intention; PR, Perceived Restorativeness.

#### 4.6.2 Sufficiency analysis of condition configuration

This study further employs fsQCA method to examine the synergistic influence mechanism of multiple antecedent variables, ED, PS, EE, and PR, on BI from a configuration perspective. Results indicate multiple convergent pathways leading to either high or low BI, with no single variable constituting a necessary condition for either outcome.

As shown in Table 15, both the configuration leading to high BI (Configuration 1) and the configuration leading to low BI (Configuration 2) exhibit Overall Consistency scores exceeding 0.85 criterion (0.894 and 0.889, respectively). This indicates that these condition combinations possess sufficient explanatory power for the outcome, serving as sufficient conditions for generating high/low BI. Both configurations exhibit Raw Coverage exceeding 0.55 (0.554 and 0.550, respectively), indicating they can explain approximately 55% of high/low BI cases, demonstrating strong empirical relevance.

**Table 15:** Analysis results of sufficient condition configuration.

Condition variables	High BI	Low BI
	Configuration 1	Configuration 2
ED	⊗	●
PS	●	⊗
EE	●	⊗
PRS		
Raw coverage	0.553566	0.549527
Unique coverage	0.553566	0.549527
Overall coverage	0.553566	0.549527
Overall consistency	0.893525	0.889404

● (large solid dots) indicating the presence of core conditions, ⊗ (large round forks) indicating the absence of core conditions, blanks indicating that the presence or absence of the condition has no effect on the results. ED, Environmental Density; PS, Perceived Safety; EE, Emotional Experience; BI, Behavioral Intention; PR, Perceived Restorativeness.

Regarding core condition distributions, both ED, PS, and EE appear as “core condition absent” (⊗) in both high-BI and low-BI configurations, while PR is blank (irrelevant). This indicates that no single variable serves as the core driver determining BI levels; their effects are highly contingent on the combined states of other variables. This finding complements SEM results: SEM reveals net effects and linear causality among variables, while fsQCA further indicates that high or low BI arises from complex interactions among multiple factors. Different variable combinations can yield identical outcomes, reflecting the “multiple concurrent causation” and “equivalence” characteristics of behavioral intention formation mechanisms.

The fsQCA findings also highlight the principle of causal asymmetry, indicating that the presence of high BI cannot be inferred from the absence of low BI, and vice versa. Different combinations of ED, PS, EE, and PR can lead to the same behavioral outcome, demonstrating equifinality in tourist decision-making. This configuration logic suggests that behavioral intentions arise not from single dominant predictors but from holistic patterns of conjunctural and mutually reinforcing conditions. Such asymmetry and conjunctural causation provide theoretical insights into visitors’ behavior that cannot be captured by SEM alone.

In summary, fsQCA analysis deepens the understanding of the complex origins of behavioral intention at the configuration level, indicating that the synergistic mechanisms of multiple variables must be examined holistically rather than isolating the influence of individual factors.

### 4.7 Qualitative validation

To further enhance the robustness of quantitative findings and uncover underlying contextual logic, this study conducted semi-structured interviews at representative night-time tourist attractions in Dalian and Chengdu of China (e.g., Dalian Xinghai Plaza, Xinghai Park, Zhongshan Plaza, Chengdu’s Kuan Zhai Alley, Jinli Ancient Street, and Fuqin Night Market). Participants spanned diverse genders, ages, and occupational backgrounds, with ages predominantly concentrated between 20 and 40 years old. Occupations included students, corporate employees, civil servants, and self-employed individuals. Through manual induction and thematic analysis, the narratives largely corroborated the linear path effects of SEM and the multi-path configuration results of fsQCA, while revealing nuanced differences not captured by quantitative analysis.

#### 4.7.1 Qualitative validation of SEM findings

Interview results broadly corroborated key conclusions from the SEM path analysis. First, high Environmental Density (ED) significantly reduced Perceived Safety (PS). One female respondent (28, teacher, Chengdu Kuan Zhai Alley) stated: “… *too crowded, it feels chaotic and unsafe, especially in narrow alleys with dim lighting. I’m worried about getting my things stolen*.” Similar descriptions emerged from Dalian respondents. A male respondent (33, office worker, Dalian Xinghai Square) remarked: “*Stalls are packed so tightly, people shoulder-to-shoulder, it’s hard to walk. I’m constantly thinking about how to protect my wallet*.”

Second, heightened security significantly boosts positive Emotional Experiences (EE). For instance, a female respondent (30, civil servant, Dalian Zhongshan Square) mentioned: *“The square is exceptionally well-lit, and there are security guards nearby. I feel reassured and completely relaxed.”* Conversely, when security is lacking, emotional experiences diminish. A male respondent (25, graduate student, Chengdu Fuqin Night Market) remarked: *“The dim lighting and crowded alleys made me feel tense, I could not enjoy browsing at all.”* Furthermore, positive emotions drive behavioral intentions. A female respondent (21, university student, Chengdu Twin Towers Light Festival) shared: *“The lights were breathtaking that night. I took tons of photos for my social media and will definitely return, this time with friends.”*

Finally, Perceived Restorativeness (PR) served as a buffer in high-density settings. A male respondent (35, freelancer, Dalian Xinghai Park) mentioned: *“Even though it was crowded, there were quiet corners to rest in, so it did not feel as oppressive.”* These narratives align strongly with SEM findings: ED indirectly influences BI through PS and EE, while PR mitigates the adverse effects of high density.

#### 4.7.2 Qualitative validation of fsQCA findings

The fsQCA analysis revealed that high Behavioral Intention (BI) is driven not by a single variable but triggered by multiple equivalent pathways. Interviews similarly exhibited this “multiple causes, single effect” characteristic. First, some respondents emphasized the combined effect of security and positive emotions. A female respondent (28, civil servant, Chengdu Jinli Ancient Street) stated: *“Although there are many tourists, the order is well-maintained, making me feel safe. Combined with the beautiful lighting and pleasant atmosphere of the ancient street, I will return next time.”* Another male respondent (31 years old, IT professional, Dalian Xinghai Square) remarked: *“It is indeed crowded, but the entire environment is managed well, so I never feel unsafe. The experience was enjoyable, and I would recommend it to friends.”*

Second, the perception of restorative qualities plays a compensatory role under high density. A female respondent (24, Master’s student, Kuan Zhai Alley, Chengdu) mentioned: *“Even though the alley is crowded, there are small teahouses and quiet courtyards where you can sit for a while. It feels like you can recharge, so it’s not too oppressive. I’d still be willing to come back.”*

However, paths leading to low behavioral intent were also confirmed in the interviews. fsQCA analysis indicates that “high density + low sense of security + low emotional experience” often leads to low BI. A female respondent (40, self-employed, Chengdu Fuqin Night Market) stated: *“It’s too crowded, and I always feel unsafe. Even though the goods are good, I worry about my wallet and cellphone. I did not feel like shopping and eventually did not want to come back.”* Some interviews also revealed the context-dependence emphasized by fsQCA. A male respondent (27, graduate student, Dalian Zhongshan Square) remarked: *“In festival events, large crowds make me feel lively and excited, but in narrow alleys, the same crowds make me feel oppressive and unsafe.”*

In summary, qualitative validation not only aligns with fsQCA’s high-low BI configuration results but also reveals differentiated manifestations of different condition combinations in specific contexts: high BI may stem from the combination of high security and positive emotions, or from the compensatory effect of perceived restorative experiences; conversely, low BI often arises from the combination of high density coupled with low security and negative emotions. Overall, the qualitative data both provide intuitive support for the linear pathways identified by SEM and vividly corroborate the nonlinear configurations revealed by fsQCA. The former exemplifies the “single pathway” mechanism, while the latter unveils the complex characteristics of “multiple pathways and equivalence.” Complementing each other, these approaches collectively demonstrate the multi-layered causal logic linking Environmental Density, Perceived Safety, Emotional Experience, Perceived Restorativeness, and Behavioral Intention within the night-time tourism context. This further enhances the external validity and theoretical explanatory power of the research conclusions.

## 5 Discussion

### 5.1 Key findings

This study examines how Environmental Density (ED) influences visitors’ Behavioral Intentions (BI) through Perceived Safety (PS) and Emotional Experience (EE), with EE conceptualized as a second-order affective construct integrating Joy, Love, and Positive Surprise. Based on SEM results, ED exerts both a direct negative effect on BI and an indirect effect through the sequential pathway “ED → PS → EE → BI.” This chain demonstrates that cognitive safety evaluations serve as the prerequisite for generating integrated emotional responses, which in turn shape visitors’ behavioral intentions. Furthermore, Perceived Restorativeness (PR) significantly buffers the negative impact of ED on PS, highlighting the protective function of restorative environmental features in crowded night-time settings.

Beyond linear effects, the fsQCA results reveal multiple configuration paths leading to high BI. One configuration emphasizes the joint presence of high PS and high EE, while another shows that even under high ED, visitors may still report strong BI when PR is high. Low BI configurations typically combine high ED with low PS and low EE. Field interview data further contextualize these findings: high density may be perceived as lively and stimulating in open, well-lit, or festive environments, whereas the same level of density can induce unease or insecurity in narrow, poorly lit, or spatially constrained settings. Importantly, the configurational solutions associated with high behavioral intention and those leading to low behavioral intention are not mirrored opposites of each other, indicating clear causal asymmetry. This pattern highlights that the presence or absence of environmental density may play qualitatively different roles across alternative configurations, thereby supporting the principles of equifinality and asymmetric causation beyond net-effect logic.

### 5.2 Theoretical contributions

This study offers four significant theoretical contributions.

First, this study extends SRT by identifying environmental density as a salient environmental stressor in night-time tourism contexts. While prior SRT research has predominantly focused on natural, low-density, and daytime environments, this study demonstrates how crowd-related stressors at night undermine perceived safety, shape emotional responses, and ultimately influence tourists’ behavioral intentions. Thereby specifying a boundary condition for SRT in socially dense nocturnal environments.

Second, this study shows that Perceived Safety and Emotional Experience form a sequential cognitive-affective mechanism consistent with the SOR framework and Affective Events Theory (AET). A key contribution lies in conceptualizing Emotional Experience as a second-order construct integrating Joy, Love, and Positive Surprise, offering a more holistic explanation of how affect shapes behavioral intention compared with traditional discrete-emotion approaches.

Third, this study validates the applicability of Attention Restoration Theory (ART) in high-density night-time tourism environments. PR is shown to act as a protective factor that buffers safety threats under crowding, broadening ART’s theoretical boundaries to include dynamic and socially dense tourism settings.

Fourth, methodological contributions arise from the combined application of SEM, fsQCA, and qualitative interviews. SEM uncovers primary linear pathways, fsQCA reveals multiple equifinal causal configurations, and interviews contextualize these mechanisms, collectively offering a robust multi-method research paradigm for understanding complex environment-psychology-behavior relationships.

### 5.3 Practical implications

The findings offer multifaceted insights for managing night-time tourism destinations.

First, effectively controlling visitor density is essential. Real-time monitoring, flexible flow regulation, reservation systems, and time-slot ticketing can help prevent overcrowding and distribute visitors more evenly. Because Environmental Density in this study comprises both human crowding and spatial crowding, management strategies should differentiate between the two. Human crowding can be alleviated through dynamic visitor flow control, peak-time scheduling, and spatial dispersal measures. Spatial crowding, in contrast, requires physical design interventions, such as widening bottleneck areas, improving circulation layouts, enhancing sightlines, and strengthening lighting in narrow or enclosed pathways, to reduce perceived enclosure and movement constraints. Addressing human and spatial density separately enables more precise improvement of perceived safety and emotional experience.

Second, a sense of security is a core psychological mechanism influencing visitor emotions and behaviors. Therefore, measures such as improving night-time lighting, increasing visible security personnel, and optimizing guidance systems are necessary to enhance visitors’ trust in the environment.

Third, this study highlights the role of restorative perception in mitigating the adverse effects of high density. This suggests that night-time tourism development should not solely pursue “bustle” and “atmosphere,” but also provide quiet, comfortable micro-spaces, such as small rest areas, natural elements, and soft lighting environments, allowing visitors to achieve psychological restoration even within crowded settings. Fourth, night-time tourism settings exhibit diversity. High density in open spaces like festival plazas may foster positive social interaction and liveliness, whereas narrow alleys or poorly lit areas are more likely to induce anxiety and insecurity. Consequently, differentiated management strategies tailored to specific locations are essential.

Finally, emphasis should be placed on conveying messages of safety and comfort through guides, staff, and intelligent service systems. This helps visitors better identify and utilize restorative resources within the environment, thereby enhancing overall emotional well-being and behavioral intentions. In addition, because Emotional Experience functions as an integrated second-order construct, destination managers should prioritize enhancing visitors’ overall affective states rather than isolated emotional cues. This can be achieved through immersive lighting design, atmospheric music, and coherent visual storytelling.

### 5.4 Limitations and future directions

Despite theoretical and methodological innovations, this study has limitations. First, the cross-sectional survey design fails to capture temporal dynamics in visitor perceptions and behaviors, which future longitudinal tracking or experimental designs could address. In addition, the use of a China-only sample limits the generalizability of the findings. Night-time tourism environments and density perceptions may vary across cultural, social, and infrastructural contexts, and future studies should examine whether the proposed mechanisms hold in other destinations.

Second, the study focused on five core variables, Environmental Density, Perceived Safety, Emotional Experience, Perceived Restorativeness, and Behavioral Intention, without incorporating potentially important factors such as place attachment, cultural authenticity, and trust. Future research could develop more complex theoretical models to deepen the explanation of visitor psychology and behavior.

Third, this study primarily relies on self-reported data, which may introduce social desirability bias. Future research could integrate multi-source evidence such as visitor behavioral trajectories, big data analysis, and physiological measurements to enhance the objectivity and robustness of findings.

Finally, while the integration of SEM and fsQCA provides a powerful tool for revealing linear and nonlinear mechanisms, there remains room for further methodological expansion. Future efforts could explore longitudinal configurational analysis, multimodal data integration, or cross-method validation to capture the complexity of environment-psychology-behavior relationships in night-time tourism more comprehensively. Future research could also deepen the conceptualization of Emotional Experience by testing alternative hierarchical structures or examining cross-cultural invariance of the second-order model.

## 6 Conclusion

This study examined how Environmental Density (ED) shapes tourists’ Behavioral Intentions (BI) in night-time tourism through Perceived Safety (PS) and Emotional Experience (EE), with Perceived Restorativeness (PR) as a moderator. Using 653 valid responses and integrating SEM, fsQCA, and field interviews, the results reveal a layered mechanism linking environmental cues, cognitive appraisal, emotional responses, and behavioral outcomes. ED decreases BI both directly and indirectly through the sequential pathway “ED → PS → EE → BI,” with EE, validated as a second-order construct, becoming influential only after safety evaluations, in line with SOR and Affective Events Theory. PR mitigates the negative impact of ED on PS, underscoring the importance of restorative environmental features in crowded night-time settings. fsQCA further uncovers multiple configuration paths to high and low BI, while interviews show that the meaning of density varies by spatial context, appearing lively in open well-lit spaces but unsafe or oppressive in narrow or dim areas.

Theoretically, this study integrates SOR, AET, and ART to clarify how cognition and emotion jointly mediate environmental effects and demonstrates the conceptual value of modeling EE as a higher-order construct. Methodologically, it illustrates how SEM and fsQCA together capture both linear mechanisms and configurational complexity. Practically, the findings recommend managing human and spatial density, strengthening safety infrastructure, and embedding restorative design elements to support positive affect and behavioral intentions.

Overall, this study advances understanding of environment-cognition-emotion-behavior mechanisms in night-time tourism and provides actionable guidance for enhancing visitor well-being and destination competitiveness.

## Data Availability

The original contributions presented in the study are included in the article/supplementary material, further inquiries can be directed to the corresponding author.
